# Developing ethical standards for dissemination and implementation research: a roadmap for consensus and guidance

**DOI:** 10.1186/s43058-023-00514-3

**Published:** 2023-11-06

**Authors:** Emmanuel K. Tetteh, Elvin H. Geng, Mark D. Huffman

**Affiliations:** 1 Office of Health Information and Data Science, Washington University School of Medicine in St. Louis, 660 S Euclid Ave, St. Louis, MO 63130 USA; 2 Division of Infectious Disease, Washington University School of Medicine in St. Louis, St. Louis, MO USA; 3 Cardiovascular Division and Global Health Center, Washington University School of Medicine in St. Louis, St. Louis, MO USA; 4 The George Institute for Global Health, University of New South Wales, Sydney, Australia

**Keywords:** Bioethics, Adverse events, Informed consent, Unintended consequences, Dissemination and implementation research

## Abstract

**Background:**

As a relatively new field, dissemination and implementation research has not been included as a separate study design category for ethical consideration compared with clinical and social/behavioral research, yet it should be based on unique study designs, targets of intervention, and corresponding risks.

**Main text:**

Research teams conducting dissemination and implementation research have raised important questions related to the responsible conduct of research such as collecting informed consent, site monitoring, identifying and mitigating risks of unintended consequences, and adverse event ascertainment and reporting in dissemination and implementation research. In this commentary, we highlight the need for guidance and consensus standards on ethical issues in dissemination and implementation research and describe some ethical domains and relevant questions in dissemination and implementation research. Additionally, we propose a process for conceptual development and a research agenda to create consensus standards for the responsible conduct of research for dissemination and implementation research.

**Conclusion:**

Thorough research is needed to understand the depth of ethical issues in dissemination and implementation research. A consensus-seeking process will be needed to develop new bioethical standards that carefully identify, measure, and mitigate unintended consequences in dissemination and implementation research.

Contributions to the literature

- There is incomplete guidance on how to define risk based on study design, particularly in late-stage translational, dissemination and implementation research
- This article highlights the need for new risk-based bioethical standards, including those related to informed consent, site monitoring, unintended consequences, and adverse event ascertainment and reporting using routinely collected health data.
- To respond to the National Heart, Lung, and Blood Institute’s call for the creation of consensus standards for dissemination and implementation research, we propose a roadmap to develop a new framework and create consensus standards for the responsible conduct of research for dissemination and implementation research.

## Background

Upholding ethical standards in research is essential to ensure that the rights and welfare of human subjects are protected. Risk-based federal and local Institutional Review Board (IRB) standards have been carefully developed for social, behavioral, and biomedical clinical trials based on the Common Rule [1]. These risks vary in terms of their probability and magnitude of potential harm or injury, including physical, psychological, social, or economic, according to the Office for Human Research Protection [2]. However, there is incomplete guidance on how to define risk based on study design, particularly in late-stage translational, dissemination and implementation research. For example, phase I-IV clinical trials of drugs and devices use common coding structures to identify and ascertain adverse events, such as the Medical Dictionary for Regulatory Activities, which was adopted by the International Council for Harmonization of Technical Requirements for Pharmaceuticals for Human Use in 1994 [3]. Social and behavioral research studies have risks that are often more variable and difficult to predict yet are often considered “no more than minimal.” However, how should risks be considered for dissemination and implementation research studies, which seek to increase the uptake of evidence-based interventions?

In this commentary, we seek to (1) highlight the need for guidance and consensus standards on ethical issues in dissemination and implementation research, (2) describe ethical domains and relevant questions in dissemination and implementation research, and (3) propose a roadmap to develop a new framework and create consensus standards for the responsible conduct of research for dissemination and implementation research.

## Main text

### Need for guidance on ethical issues in dissemination and implementation research

Dissemination and implementation research is a growing field within the translational sciences, health systems, and policy research arenas, which is yet to be included as a separate study design category for ethical consideration compared with clinical and social/behavioral research. However, dissemination and implementation research spans both through its study designs (e.g., stepped wedge), targets of intervention (e.g., health systems, communities, policies), and corresponding risks (e.g., clinician burnout). The Common Rule defines minimal risk as “that which is ordinarily encountered in daily life or in the routine medical, psychological, or educational examinations, tests, or procedures of the general population” [1]. Dissemination and implementation research studies seek to increase the uptake and spread of evidence-based interventions, including across varied populations who may bear a disproportionate burden of disease at baseline, and thus arguably could inherently have only minimal risk to patients and other research participants.

The National Institutes of Health has increasingly issued multi-institute funding announcements to encourage research that focuses on dissemination and implementation research, including funding > $100 million in multi-site consortia since 2017 [4]. The growing investments have raised on-the-ground questions related to the responsible conduct of research such as collecting informed consent, site monitoring, unintended consequences, and adverse event ascertainment and reporting using routinely collected health data [5, 6]. An expert review report on the ethics of health policy and system research, including dissemination and implementation research noted that “while there is some literature on ethical issues in health systems and policy research, it is not reflective of the breadth or depth of potential ethical issues or comparable to the volume and quality of ethics scholarship done in other fields such as clinical research” [6]. In 2020, the National Heart, Lung, and Blood Institute called for the creation of consensus standards for dissemination and implementation research [5]. Research teams need guidance for application into practice according to the specific populations and conditions, contexts, and implementation strategies under study.

### Ethical considerations in dissemination and implementation research

Defining and understanding the ethical considerations of dissemination and implementation research is necessary to guide the development of risk-based consensus standards. Dubois and Prusazyk outlined common, relevant questions in dissemination and implementation research that are based on the principles of the Belmont Report and can serve as useful starting points [7]: (1) Is it human subjects research? (2) Who are the research participants and who should provide informed consent? (3) Is equipoise necessary? and (4) How can scientific rigor be protected in routine-care settings? Illustrative examples will assist in comprehending the differences in these ethical considerations between dissemination and implementation research and the conventional approach in clinical/biomedical trials. These insights have been elaborated upon in the preceding paragraphs, accompanied by a summary table outlining the considerations and their applications in biomedical and dissemination and implementation research (Table 1).

1. Is it human subjects research?
  Research studies are classified as human subject research when they entail identifiable private information or direct interaction or intervention with individuals, aiming to advance generalizable knowledge [8]. In biomedical research, this definition is applied relatively clearly and consistently. For instance, in the Quadruple Ultra-Low-Dose Treatment for Hypertension (QUARTET) trial, 591 participants were randomly assigned to receive a quadpill or regular therapy, and their blood pressure data were collected and analyzed [9]. Since the QUARTET trial involved participant enrollment, treatment assignment, and data collection, it qualified as human subject research and underwent review by a research ethics committee. However, determining whether a study qualifies as human subject research within the context of dissemination and implementation studies can be a nuanced process, even when there is no private identifiable information or direct interaction or intervention with individuals in the study. Furthermore, studies focused on improving healthcare outcomes in an organization may sometimes be categorized as quality improvement projects, thereby exempting them from IRB oversight, even when data collection occurs at the patient or cluster level. The level of IRB oversight for quality improvement studies is inconsistent and varies widely [10]. For instance, the Kaiser Permanente Northern California hypertension program [11], a quality improvement program involving over 300,000 participants, was exempt from IRB review, while a similar quality improvement study in acute myocardial infarction patients—the Acute Coronary Syndrome Quality Improvement in Kerala, India (ACS QUIK) trial—required approval from multiple IRBs and ethics boards [12].
2. Who are the research participants and who should provide informed consent?
  Defining research participant roles and securing their informed consent are essential aspects of research practice. Guidelines in biomedical trials establish the providers of informed consent, specify the timing, and determine the depth of consent required. For instance, in the QUARTET trial, enrolled participants in treatment and control groups provided written informed consent, detailing potential risks and harms, before study procedures commenced [9]. Determining research participants, informed consent providers, and evaluating potential risks in dissemination and implementation research involves a nuanced approach. Illustrating this, the Hypertension Treatment in Nigeria (HTN) Program adopts a blend of strategies from Kaiser Permanente Northern California hypertension program intervention components and the World Health Organization’s HEARTS (Healthy-lifestyle counseling; Evidence-based treatment protocols; Access to essential medicines and technology; Risk-based cardiovascular disease management; Team-based care; Systems for monitoring) technical package to integrate cardiovascular care in primary care settings [13, 14]. These strategies encompass patient, clinician, and system levels, thereby presenting a different need for informed consent based on each of the level of risk participants are exposed to. For instance, is it necessary to receive informed consent from all patients who receive care at the participating health centers and from the clinicians who provide care at participating research sites to implement this multilevel bundle? While this may seem impractical, some cadres may be exposed to greater risks than others in such large-scale multilevel implementation trials [15].
3. Is equipoise necessary?
  Clinical equipoise refers to genuine uncertainty among clinical investigators regarding the comparative therapeutic merits of trial arms [16]. While establishing equipoise is generally challenging in research, it is more straightforward in biomedical trials. For example, the Preexposure Prophylaxis Initiative (iPrEx) trial [17] exhibited clear equipoise between a new treatment modality (PrEP—preexposure prophylaxis) and a placebo, creating genuine uncertainty among investigators and the medical community.
  However, in dissemination and implementation studies, justifying equipoise for evidence-based interventions can be challenging. Consider the Preexposure Prophylaxis Optimization Intervention (PrEP-OI) study, which aimed to enhance PrEP prescriptions through two implementation strategies—a PrEP coordinator and an adapted web-based management tool [18]. The already well-studied merit of PrEP usage makes equipoise of this evidence-based intervention unnecessary to justify. Nonetheless, significant variability may exist in the implementation strategies required for successful uptake of the evidence-based intervention in the study population or context.
  Another concern related to justifying equipoise, in accordance with the ethical guidance outlined by the Ottawa Statement on the Ethical Design and Conduct of Cluster Randomized Trials [19], is ensuring that research participants in both the intervention and control arms of a research trial benefit from evidence-based interventions and treatments when the benefits are known to be superior. Adaptive designs, such as the stepped-wedge approach, are becoming increasingly common in dissemination and implementation studies and have the potential to address this ethical concern [20, 21]. A stepped-wedge study design introduces evidence-based interventions gradually over time, necessitating ongoing data collection and adaptation. This dynamic process, however, poses ethical challenges for investigators and ethical review boards striving to align with the evolving nature of study procedures [20, 21].
4. How can scientific rigor be protected in routine care settings?
  Maintaining scientific rigor in dissemination and implementation research presents distinctive challenges compared to clinical trials of new treatment drugs in biomedical research. While both biomedical and dissemination and implementation research prioritize methodological precision, dissemination and implementation research mostly operates within the complexities of routine care settings. In clinical drug trials, researchers can typically control variables, monitor outcomes, and standardize conditions. In contrast, dissemination and implementation research involves implementing interventions across diverse healthcare settings, requiring adaptation to various contexts – contexts that are typically challenging to control or standardize. Ensuring uniformity and internal validity becomes complex amidst such variability.
  The engagement of multiple stakeholders in dissemination research adds another layer of complexity. Unlike biomedical trials primarily focused on participant outcomes and safety, dissemination and implementation research involves healthcare providers, administrators, policymakers, and patients. Balancing scientific rigor with the practical considerations of diverse stakeholders becomes crucial. Moreover, the long-term sustainability of interventions and adaptability to evolving contexts pose unique challenges. While biomedical trials often have defined durations, dissemination and implementation research studies seeking successful uptake of evidence-based interventions need to account for changing policies, personnel shifts, and evolving patient demographics over extended periods.
  Innovative methodologies, such as adaptive trial designs, data management systems, and interdisciplinary collaborations, have become pivotal in maintaining scientific rigor while navigating the practical intricacies of routine care implementation [22]. Consequently, the intricate nature of dissemination and implementation research, with its challenges and complexities, often leaves ethical review boards seeking guidance on how to ethically evaluate such studies, given their unique considerations [5–7].

**Table 1:** Ethical considerations comparison between biomedical and dissemination and implementation research

**Ethical considerations**	**Biomedical research**	**Dissemination and implementation research**
**Is it human subjects’ research?**	Individuals often serve as subjects, with focus on safety and individual outcomes	Subjects can be individuals, groups, or entire health systems. Ethical review may be needed for interventions affecting broader systems
	Ethical review often focuses on individual participant rights and well-being	Ethical review boards may need to assess impact on communities, organizations, and health delivery systems. Waiving review for quality improvement should be carefully considered
**Who should provide informed consent?**	Consent typically obtained from individual participants	Consent considerations extend to healthcare providers, administrators, and patients. Scope includes potential impact on systems and practices in implementation
	Consent form outlines study purpose, procedures, risks, and benefits for individuals	Consent may cover changes at the system level, understanding potential effects on multiple stakeholders
**Is equipoise necessary?**	Control groups often used to compare new interventions	Balancing equipoise can involve changes in practices, necessitating ethical reasoning for control groups. Uncertainty in scientific merit is important, but implementation context adds complexity
	Equipoise considers balancing risks and benefits for individual participants	Risk-benefit assessment includes potential system-level and societal impact, as well as individual well-being
**How can scientific rigor be protected in routine care settings?**	Rigor focuses on experimental design, data collection, and analysis	Rigor includes evaluating how interventions integrate into real-world contexts. Added challenge of assessing system-level outcomes
	Focus on addressing potential biases from study design and analysis	Consideration of biases extends to the impact of biases on healthcare delivery and system-level outcomes. Balancing scientific rigor with the practical considerations of diverse stakeholders is challenging.

### Roadmap for consensus standards development

Defining ethical considerations and addressing general questions is a crucial initial step, providing valuable groundwork. Moreover, research teams need more specific guidance for application into practice according to the specific populations and conditions, contexts, and implementation strategies under study. To respond to the National Heart, Lung, and Blood Institute’s call for consensus standards [5], we propose a roadmap for their development.

1. Delphi approach to modify existing ethical frameworks
  First, consensus among dissemination and implementation researchers and stakeholders including ethical review boards, funding agencies, patients, or clients, etc., using a multi-stage modified Delphi approach can be used to modify existing ethical frameworks into dissemination and implementation research. Field-specific questions related to the scope and methods of monitoring, reporting, and responding to adverse events, defining minimal risk and other constructs relevant to waiving or modifying informed consent must be carefully elaborated in this consensus seeking process.
2. Map implementation strategies to unintended consequences
  Second, Pullman and colleagues identified 13 categories of potential unintended consequences, or ripple effects, of implementation strategies to promote uptake of evidence-based interventions for children’s mental health services [23]. Categories of positive or negative unintended consequences can serve as a guide upon which implementation strategies can be mapped with domain definitions related to feasibility, frequency, impact, timeframe, measurement, and mitigation.
3. Train research teams on consensus-driven ethical framework
  Third, research teams conducting dissemination and implementation research will require training on the use of the consensus-driven framework to identify and define the scope of waiving or obtaining informed consents in dissemination and implementation research, to develop methods of ascertainment, monitoring, and reporting of unintended consequences, and to develop strategies to mitigate risks posed by unintended consequences, while maintaining the scientific rigor of studies. The federally-funded Heart, Lung, and Blood Co-morbiditieS Implementation Models in People Living with HIV (HLB SIMPLe) Alliance includes six research teams pursuing late-stage cardiovascular dissemination and implementation research to integrate cardiovascular services into care among individuals living with HIV in Botswana, Mozambique, Nigeria, South Africa, Uganda, and Zambia. This alliance offers one of many potential opportunities to contribute to the development of a consensus-driven framework and standards to improve the responsible conduct and reporting of dissemination and implementation research. Furthermore, collaborations between other consortia on the implementation of the consensus-driven standards will be needed to achieve the long-term goal of influencing updated regulatory standards that incorporate distinct features of dissemination and implementation research.

## Conclusions

As a relatively new field, dissemination and implementation research has not been included as a separate study design category for ethical consideration compared with clinical and social/behavioral research. Rigorous research and a consensus-seeking process are needed to develop new risk-based bioethical standards, including those related to informed consent, site monitoring, identifying and mitigating risks of unintended consequences, and adverse event ascertainment and reporting in dissemination and implementation research studies.

## Data Availability

Not applicable.

## Abbreviations

CVD: Cardiovascular disease
EBI: Evidence-based intervention
HIV: Human immunodeficiency virus
HLB-SIMPLe: Heart, Lung, and Blood Co-morbiditieS Implementation Models in People Living with HIV
IRB: Institutional Review Board
PrEP: Preexposure prophylaxis

## References

[1] National Research Council of the National Academies. Proposed revisions to the common rule for the protection of human subjects in the behavioral and social sciences. National Academies Press (US). 2014. https://www.ncbi.nlm.nih.gov/books/NBK217976/?report=classic. Accessed 24 Jan 2023. 25032406

[2] Office for Human Research Protection. Institutional Review Board Guidebook. (1993). https://www.hhs.gov/ohrp/register-irbs-and-obtain-fwas/index.html. Accessed 24 Jan 2023.

[3] Medical Dictionary for Regulatory Activities. https://www.meddra.org/. Accessed 24 Jan 2023.

[4] Sekar K. National Institutes of Health (NIH) Funding: FY1996-FY2023. Congressional Research Services 2022; R43341. https://crsreports.congress.gov/product/pdf/R/R43341/44. Accessed 24 Jan 2023.

[5] National Heart, Lung, and Blood Institute Implementation Science Workshop Summary. https://www.nhlbi.nih.gov/events/2020/ethical-questions-implementation-science-workshop-summary. Accessed 24 Jan 2023.

[6] Hyder AA, Pratt B, Ali J, Kass N, Sewankambo N (2014). The ethics of health systems research in low- and middle-income countries: a call to action. Glob Public Health..

[7] DuBois JM, Prusaczyk B. Ethical issues in dissemination and implementation research. In: Brownson RC, Colditz GA, Proctor EK, editors. Dissemination and Implementation Research in Health: Translating Science to Practice. 2nd ed. Oxford Academic; 2017. 10.1093/oso/9780190683214.003.0004

[8] The U.S. National Archives and Records Administration. eCFR :: Title 45 The Protection of Human Subjects. https://ecfr.federalregister.gov/current/title-45/subtitle-A/subchapter-A/part-46. Accessed 2 August 2023.

[9] Chow CK, Atkins ER, Hillis GS (2021). Initial treatment with a single pill containing quadruple combination of quarter doses of blood pressure medicines versus standard dose monotherapy in patients with hypertension (QUARTET): a phase 3, randomised, double-blind, active-controlled trial. Lancet..

[10] Nerenz DR, Stoltz PK, Jordan J (2003). Quality improvement and the need for IRB review. Qual Manag Health Care..

[11] Jaffe MG, Lee GA, Young JD, Sidney S, Go AS (2013). Improved blood pressure control associated with a large-scale hypertension program. JAMA.

[12] Huffman MD, Mohanan PP, Devarajan R (2017). Acute coronary syndrome quality improvement in Kerala (ACS QUIK): rationale and design for a cluster-randomized stepped-wedge trial. Am Heart J.

[13] Baldridge AS, Aluka-Omitiran K, Orji IA (2022). Hypertension Treatment in Nigeria (HTN) Program: rationale and design for a type 2 hybrid, effectiveness, and implementation interrupted time series trial. Implement Sci Commun.

[14] World Health Organization. HEARTS technical package for cardiovascular disease management in primary health care: risk based CVD management. World Health Organization, Geneva; 2020. https://www.who.int/publications/i/item/9789240001367. Accessed 24 Jan 2023.

[15] Kruse GR, Chapula BT, Ikeda S (2009). Burnout and use of HIV services among health care workers in Lusaka District, Zambia: a cross-sectional study. Hum Resour Health..

[16] Freedman B (1987). Equipoise and the ethics of clinical research. N Engl J Med..

[17] Grant RM, Lama JR, Anderson PL (2010). Preexposure chemoprophylaxis for HIV prevention in men who have sex with men. N Engl J Med..

[18] Ming K, Shrestha I, Vazquez A (2020). Improving the HIV PrEP continuum of care using an intervention for healthcare providers: a stepped-wedge study protocol. BMJ Open..

[19] Weijer C, Grimshaw JM, Eccles MP (2012). The Ottawa statement on the ethical design and conduct of cluster randomized trials. PLoS Med..

[20] Prost A, Binik A, Abubakar I (2015). Logistic, ethical, and political dimensions of stepped wedge trials: critical review and case studies. Trials..

[21] Hemming K, Haines TP, Chilton PJ, Girling AJ, Lilford RJ (2015). The stepped wedge cluster randomised trial: rationale, design, analysis, and reporting. BMJ..

[22] Geng EH, Peiris D, Kruk ME (2017). Implementation science: relevance in the real world without sacrificing rigor. PLoS Med.

[23] Pullmann MD, Dorsey S, Duong MT (2022). Expect the unexpected: a qualitative study of the ripple effects of children’s mental health services implementation efforts. Implement Res Pract..

